# Cardiac Multidetector Computed Tomography: Basic Physics of Image Acquisition and Clinical Applications

**DOI:** 10.2174/157340308785160615

**Published:** 2008-08

**Authors:** Dianna M.E Bardo, Paul Brown

**Affiliations:** Director of Cardiac Radiology, Oregon Health and Science University, 3181 SW Sam Jackson Park Rd – CR 135, Portland, OR 97239, USA

**Keywords:** Cardiac CT, coronary artery MDCT, multidetector CT, dual source CT.

## Abstract

Cardiac MDCT is here to stay. And, it is more than just imaging coronary arteries. Understanding the differences in and the benefits of one CT scanner from another will help you to optimize the capabilities of the scanner, but requires a basic understanding of the MDCT imaging physics.

This review provides key information needed to understand the differences in the types of MDCT scanners, from 64 – 320 detectors, flat panels, single and dual source configurations, step and shoot prospective and retrospective gating, and how each factor influences radiation dose, spatial and temporal resolution, and image noise.

## INTRODUCTION

Computed axial tomography (CAT) scan or CT has developed from imaging 10 mm thick slices of the motionless brain in a period of several minutes, to multidetector CT (MDCT) which continues to advance today to imaging the beating heart in less than 10 seconds, using sub millimeter slice thickness.

The future development of MDCT is limited only by imagination and the mechanical limitations of the scanner. Technical invention and innovation has not only advanced well-established imaging indications but has directed medical imaging into new directions. In order to best utilize these technical advances, perhaps direct the manufacturers toward clinically inspired imaging needs and to make the most of current technology the user must be knowledgeable about the mechanics of the various types of CT scanners, the physics of radiation dose and delivery, and the physics of image acquisition. The advances in MDCT scanning have resulted in numerous variations of 64-320 channel MDCT scanners being available on the market and in use throughout the world. In order to best serve an individual patient population and to achieve your imaging goals, interpreting physicians must thoroughly understand the properties and capabilities of the type of scanners in their institution.

This article reviews MDCT scanners, from 64 – 320 detectors, single and dual source configurations, flat panel detectors, step and shoot prospective and retrospective gating, and how each factor influences radiation dose, spatial and temporal resolution, and image noise.

Increased understanding of current MDCT technology is beneficial to the experienced and new user in that exam quality and patient through-put are increased and radiation dose is optimized and reduced. Further, this greater understanding will serve to inspire continued evolution of new clinical and research applications of cardiac MDCT – myocardial perfusion, myocardial and valve function and even greater spatial and temporal resolution which will have widespread application in the principles of MDCT.

## DISCUSSION

### Early CT Scanners

Single detector CT scanners were first utilized in the early 1970’s. The images produced were acquired in the axial plane as individual slices 8-13 mm in thickness. The scanner gantry had a single detector which translated around the patient as the patient table moved in the cephalocaudal (z-axis) direction. As early as 1975 there are descriptions of reconstruction techniques using axial CT data to construct coronal and sagittal image planes [1]. Second and third generation CT scanners were improved by increasing the number of detectors and by converting the translation motion of the radiation source to rotation, respectively, resulting in exponential increases in imaging speed with improved image quality. Utilizing these data image reconstruction has also been revolutionized. Standard two dimensional (2D) images now are beyond axial, sagittal and coronal planes to include off axis and curved planar images. Three dimensional (3D) surface rendered images are now commonly displayed in motion, adding a fourth dimension (4D) [2].

## MDCT

In the 1980s 4 channel MDCT appeared in commercial use. Since that era, the addition of more imaging channels, rows or detectors has increased exponentially. Subsequent improvements in the mechanics of the scanners have resulted in faster x-ray gantry rotation, faster z-axis movement of the CT table, thinner collimation and the development of electrocardiographic (ECG) gating. Each of these advances has improved temporal and spatial resolution.

The newest commercially available MDCT scanners (seventh generation) offer the greatest degree of spatial and temporal resolution owing to up to 320 detector channels, gantry rotation times of .25 seconds and collimation as narrow as .24 mm. These parameters are important to creation of high definition coronary artery MDCT imaging and are invaluable in imaging of myocardial and valve function, pulmonary vein and aortic anatomy. 

Four major CT manufacturers were asked to provide information about MDCT scanners currently commercially available in the United States; further data were acquired from the manufacturer’s web sites http://www.gehealthcare.com/usen/ct/index.html; http://www.medical.philips.com/main/products/ct/; http://www.medical.siemens.com; http://www.medical.toshiba.com/Products/CT/).

Before discussing the mechanics of each variation in scanner construction, it is important to understand the image parameters necessary to result in high definition ECG-gated cardiac images. The basic anatomy of MDCT scanner components is shown Fig. (**1**).

## ECG-GATING

ECG gating enables acquisition of image data and image reconstruction at precise time points during the R-R interval. Image acquisition can occur throughout the R-R interval (retrospective gating) or at a predefined time point (prospective gating). The R-R interval can theoretically be divided into 100 segments for the purposes of cardiac CT. The first R wave is the zero of 0% time point at end diastole; the next R wave is the 100% time point of the first cardiac cycle and the 0% time point of the next cardiac cycle Fig. (**2**).

In prospective gating, usually referred to in catchy, proprietary terms by manufacturers, the radiation source is active only during a short segment of the R-R interval, optimally at 70% - 80% of the R-R interval when imaging the coronary arteries. No radiation or a markedly reduced radiation dose is delivered to the patient during the remainder of the R-R interval, resulting in reported dose savings approximately 80% Fig. (**3**). When using prospective gating, imaging occurs only during every other heart beat, allowing time for table motion and resetting of timing to the next R wave between images. This type of acquisition is not helical; instead, image data is acquired one slice at a time, while the patient moves incrementally in the z axis. Subsequently the images are stacked into a single sequence of image data which can be post processed in the usual manner to evaluate the coronary arteries and morphology of the heart. Functional data can not be obtained as only a short segment of the cardiac cycle has been imaged; i.e. image data is not acquired during both end systole and end diastole [3].

Using retrospective gating, image data are acquired throughout several cardiac cycles and is subsequently divided into segments based upon the time lapsed from the beginning of each R-R interval. The first R wave is the 0% time point; each subsequent R wave is the 100% time point for the previous cardiac cycle and the 0% time point for the next R-R interval. The user defines the segment of image data which will be reviewed for diagnosis. Image data can be divided and viewed in up to 100 segments or blocks of time; typical segmental reconstruction protocols divide image data into 10 or 20 equally spaced segments across the R-R interval.

Myocardial functional data can be evaluated in this manner because all segments of the cardiac cycle, particularly end diastole and end systole, are imaged. The radiation dose delivered to the patient is much higher using retrospective gating compared to prospective gating because the patient is exposed to radiation continually, throughout the scan [4]. Each manufacturer has a method for dose reduction when using retrospective gating which decreases the radiation dose during the phases of the scan which are typically suboptimal for coronary artery imaging.

Retrospective triggering, another way to time image acquisition with the cardiac cycle, is one of the methods commonly utilized in cardiac magnetic resonance imaging (CMRI). The R wave acts as a trigger to initiate image acquisition. Following the trigger a short amount of time elapses before the acquisition begins. Therefore, when viewing images in a cine mode, there may be a perceptible irregularity in the fluidity of myocardial motion. Retrospective triggering is not used in cardiac CT as the technique would have very little value in decreasing radiation dose and would induce the same artifacts of image motion.

## IMAGE RESOLUTION

### Temporal Resolution

Temporal resolution is defined as the length of time required to image an object. In order to image a moving structure with a high degree of temporal resolution, i.e., without motion artifacts, one must acquire the image faster than that structure is moving. In imaging, the gold standard for temporal resolution has been set by conventional angiography which has temporal resolution of about 20 msec [4, 5].

When considering MDCT scanners, the parameters which affect temporal resolution are speed of gantry rotation, pitch, the ability to acquire image data in a segmented fashion. Current technology allows gantry rotation times as low as 0.33 sec. Speed of gantry rotation is limited by the g forces produced by the rotating gantry upon the x-ray tube; for example at a rotation speed of .5 sec exceeds 10 g [2]. 

It is not necessary to use the data acquired in a full 360 degree rotation in order to reconstruct an image of the heart. Therefore, in a method referred to as segmenting the scan, single source MDCT scanners use the data acquired in 180 degrees of rotation (½ scan). Using a ½ scan technique, twice the image data is acquired during a full rotation or 360 degrees. This method allows faster scanning, improving the temporal resolution to 165 msec for a single source scanner [6, 7] When the scanner gantry rotates 360 degrees in 0.33 sec, the temporal resolution of the scan is 330 msec which is decreased to 33 msec when ECG gating reconstruction divides the cardiac cycle into 10 segments of the R-R interval, approaching the temporal resolution of conventional angiography.

## PITCH

Pitch for MDCT is defined as the longitudinal (z-axis) table increment per 360 degree gantry rotation / beam collimation [5, 8]. A higher pitch value indicates the table is moving in the longitudinal z-axis at a faster rate. At a greater pitch the scan is completed faster, potentially resulting in fewer motion artifacts related to breathing or voluntary motion. However, spatial resolution may suffer due to increased image noise [5]. One MDCT scanner manufacturer (Siemens) attempts to maintain adequate image resolution (noise) when pitch is increased by increasing tube current (mAs) [9].

### Spatial Resolution

Spatial resolution is defined as the ability to discern two objects as separate from one another. When referring to a radiographic image, numerous factors regulate the degree of spatial resolution achieved. These are slice thickness, collimation, reconstruction increment, filtering and field of view. Generally, though also meaningful in increasing spatial resolution, the following parameters can not be adjusted by the user; speed of tube rotation, focal spot size, detector size and the distance between the focal spot and the detector ring [10]. The gold standard for spatial resolution has been set by conventional angiography which has spatial resolution capable of less than 0.2 mm [5].

When making decisions about imaging protocols, one must consider when to favor temporal resolution over spatial resolution and vice versa. Temporal resolution is important when imaging anatomy that moves, such as the heart and coronary arteries. One should also consider how breathing motion artifact will affect image quality in patients who can not hold their breath or suspend respiration. Protocol choices such as faster scanning which improve temporal resolution will be at the expense of spatial resolution because of the increase in image noise. The opposite is true when adjusting imaging protocols in favor of optimizing spatial resolution. Reducing the speed of image acquisition in order to more clearly reveal the fine details of anatomy takes longer, therefore allowing more time for motion artifacts to occur (reduced temporal resolution). Scanning for a longer time and narrowing collimation also increase the radiation dose delivered to the patient and decreases image contrast resolution, i.e., increases image noise [5, 7].

### Image Noise

Noise in a MDCT image occurs as a consequence of variation of image contrast resolution. When measured in an area of uniform attenuation, noise, expressed in Hounsfield units (HU), is the standard deviation of the pixel values within that area Fig. (**4**). Image noise (quantum noise) is affected by the number of photons reaching the detectors, electronic noise of the system and reconstruction algorithms (kernels), gantry rotation time, pitch and slice collimation. Quantum noise is proportional to the square root of the number photons which hit the detectors and is therefore the dominant factor. Setting aside patient factors such as body habitus, tube current, slice collimation width, pitch and scan time are major factors which influence image noise over which the operator has control [7].

### 64 – 320 Channels

Each manufacturer currently offers a 64 channel MDCT scanner with ECG-gating software for cardiac imaging. Differences in imaging parameters include variations in the arrangement of the detector array; slice thickness collimation and gantry rotation times which result in slight differences in spatial and temporal resolution.

As the number of detector channels increases, in currently available scanners, to 128, 256 and 320, and with faster gantry rotation speeds, the temporal resolution (and spatial resolution) improves, [7, 11] in theory, leading to better cardiac imaging.

### Dual Source MDCT

A dual source MDCT scanner utilizes two sources of radiation and corresponding sets of detectors oriented 90 degrees from one another Fig. (**5**). Image data may therefore be acquired twice as fast as with a single radiation source and set of detectors. As mentioned earlier, with single source MDCT scanners segmented scanning techniques utilize only ½ of the gantry rotation (180 degrees) for image reconstruction. Dual source MDCT gathers this same amount of image data in ¼ of the rotation (90 degrees) [12].

The increased scanning speed of dual source MDCT results in a 50% decrease in scanning time and consequent increase in temporal resolution (83 msec) independent of HR. With dual source MDCT data used to image anatomy the width of the detectors are acquired during only one heart beat. This point is important because the motion inherent from the changing position of a coronary artery from one heart beat to another which is still perceptible with single source MDCT is eliminated. The temporal resolution is heart rate independent but does require the heart rate to be perfectly steady. HR stability is necessary so that the ECG trace can be evaluated prior to the scan, allowing the scan acquisition to be signaled accurately. If it were technically possible to increase the speed of gantry rotation, further improving temporal resolution, this necessity could be overcome [6, 7, 13].

Although it at first seems that using 2 radiation sources would double the radiation dose of a cardiac MDCT scan the radiation dose is actually cut in half – or does not exceed that of a single detector dose modulated scan. This occurs because the radiation source is turned on for less than ½ the time, narrowing the ECG pulse window [14].

### Flat Panel

Flat panel CT is theoretically the future of cardiac CT and the return to a ‘single’ detector. The detector panel, much like that utilized in C-arm fluoroscopy would allow for attaining the superior spatial resolution of cardiac catheterization. The difficulty is that the detector panel would be physically larger and heavier than the detector panels in current MDCT scanners. The mechanical limitations of current scanner technology will not allow adequate rotation speed of such a detector for cardiac imaging. Therefore temporal resolution with such a detector for CT would be inadequate to resolve cardiac motion [2].

Superior spatial resolution of flat panel CT is intriguing not only for the possible diagnostic importance, but for the possibilities of CT guided/navigated therapeutic interventions. Can you imagine performing a CT of the coronary arteries, diagnosing thrombus in the mid LAD and then advancing a micro catheter into the LAD and delivering anticoagulant into the thrombus and watching it dissolve? How about placing a coronary artery stent under CT guidance; watching the stent open and then perfecting the angioplasty results, under direct visualization?

Embarking on this path toward combined CCTA and cardiac intervention would be beneficial in that the diagnostic portion of a standard cardiac catheterization exam could be eliminated as this information would be available with the CCTA. Rapid post-processing of the CCTA data allows one to immediately know the fluoroscopic angle for optimally viewing a stenosis during the treatment procedure, without the need for multiple angiograms. Post-processed 2D and 3D images show the anatomy of the entire heart and the lumen of coronary arteries and veins, clarifying anatomical relationships and sharpening diagnostic conspicuity. Therefore, radiation dose and procedure time could be dramatically reduced. Beyond reducing patient exposure, the radiation exposure to the cardiac interventionalist, nursing and technical staff is also reduced. Shorter procedure times could potentially lower complication rates as well.

## RADIATION EXPOSURE AND DOSE

Radiation exposure is the amount of ionization of air as the result of an x-ray beam. In CT the radiation dose to each slice is measured in a quantity knowns CT dose index (CTDI), in mGy, a quantity that is calculated from the parameters of the scan and displayed on the scanner display at the time of the scan. Also displayed on the scanner is the dose length product (DLP), which is the product of the z-axis length of the scan and the CTDI, in units of mGy · cm [7, 13].

The overall risk of radiation dose is defined as the effective dose. The effective dose given by multiplying an organ dose times a weighting factor which is specific to the anatomy that has been scanned. For the heart, a factor of 0.17, [15] is specific for the thorax; DLP · 0.17 = effective dose. Most scanners provide an estimate of the CTDI at the time of the scan.

### Factors Affecting Radiation Dose

The radiation dose given in CT is controlled by the operator by adjusting the x-ray tube voltage (kVp), tube current a measure of milliamperes and scan time in seconds (mAs), table pitch, gantry rotation time and detector configuration [9]. Tube voltage values of 120 to 140 kVp are routinely used for adult cardiac MDCT. Decreasing kVp is a viable method to reduce radiation doses in pediatric patients and in young adults, especially for women in whom radiation exposure to breast tissue is of concern, for future risk of breast cancer [16]. Reducing kVp decreases radiation dose, but results in increased image noise. Tube current values can vary over a broad range; image quality is generally improved when a higher mAs is utilized. Radiation dose and image noise are directly affected by mAs [7].

As the CT table moves in the z-axis the radiation dose is imparted to the chest one slice at a time. If the table speed (pitch) is increased, the radiation dose is lower because the anatomy is exposed to the radiation beam for a shorter amount of time. Similarly as the speed of the gantry rotation increases around the patient, the radiation dose is decreased. Increasing pitch and the gantry rotation time have an often poorly understood adverse affect upon image noise [7, 9, 13]. In MDCT, the operator does not always have control over each of theses parameters. On Siemens scanners when one adjusts the pitch, in order to scan faster, the mAs is automatically increased in order to maintain low image noise [12].

As indicated earlier; retrospective ECG gating is a costly method of ECG gating for coronary artery CTA. The radiation dose delivered in a typical exam performed with a 64 detector scanner ranges between 10-15 mSv. This dose is accumulated in overlapping helical acquisitions which are acquired throughout several cardiac cycles. The detector overlapping during the image acquisition provides redundant data which are then interpolated into the final image. The amount of redundant data varies inversely with the pitch [7]. A portion of the beam as the scan begins and ends is not utilized in accumulating image data but is wasted as the gantry completes a part of the first and last rotations. This portion of the scan is referred to as over-scanning Fig. (**6**). When calculating the total effective radiation dose it is important to consider the dose delivered in the scan and the dose delivered at the edge of the scan.

Greater z-axis scan coverage achieved with the wider detector panel requires fewer rotations of the gantry be performed to image the heart. Therefore fewer overlapping helices are required, shortening image acquisition time. With 256 and 320 channels the detector array covers 80 mm [personal communication, Ami Altman, Ph.D.], to 120 mm [17] and 160 mm [personal communication, Rich Mather, Ph.D.], [18], respectively, depending upon manufacturer, allowing the entire heart to be imaged in a one or two rotations of the gantry. Although theoretically fewer gantry rotations should reduce radiation dose by limiting overlapping rotations of the gantry, [19] the CT dose index (CTDI) of CCTA scans performed with 256 and 320 detector channels is exactly the same as a scan performed with 64 detector channels, covering the same z-axis of the chest. Anticipated dose savings of 10% - 15% or more may be achieved through the use of bow tie filters and dynamic collimation techniques [personal communication, Ami Altman, Ph.D. and Rich Mather, Ph.D.] [20].

Prospective ECG gating is currently the most radiation dose efficient method for performing coronary artery MDCT. The radiation beam is on only during a very short segment of the R-R interval; with 64 detectors, the radiation dose is decreased to 3-5 mSv.

To briefly put radiation dose into perspective, it is helpful to review estimates of radiation dose exposure from common diagnostic imaging procedures and background radiation. Table **2**.

### Increasing Number of Detector Rows

With single detector CT a narrow radiation beam is emitted, with very little slice overlap, exposing one slice of tissue at a time. Therefore, as one 360 degree rotation of the radiation source occurs, one image is produced [21].

As the number of detector rows increases the radiation beam width increases into a broad cone shape from the radiation source to span the detectors. As the cone-shaped beam widens to cover a greater number of detectors, the amount of overlap which occurs during helical image acquisition results in collection of redundant data which contributes to improving spatial resolution and image resolution (noise). The redundancy of data allows one to segment the scan rotation into a partial scan, i.e., using only 180 degrees or ½ of the scan data to create the image. In order to avoid overlap of the radiation beam table speed or gantry rotation speed would have to be markedly slowed, resulting in concomitant decreases in temporal resolution [7, 9].

## CARDIAC MDCT IMAGING – PRESENT AND FUTURE

The rapid growth in cardiac MDCT is both the result of manufacturer progress and the desire of imaging physicians to utilize this technology and to inspire the manufacturer to explore further advanced imaging concepts. Current and potential future options in cardiac MDCT are discussed very briefly in the next section.

### Coronary Artery Angiography

Accepted indications for coronary artery MDCT include detection of calcium scoring, anomalous coronary artery origins, identification of myocardial bridging, detection of coronary artery disease (CAD) including soft plaque and degree of stenosis, especially in symptomatic patients with low to intermediate pre-test probability of coronary artery disease [4, 22, 23] Fig. (**7A** & **7B** & **7C**). Vascular injury such as aneurysm and dissection may also be detected and have been described in case reports [24] Fig. (**8A** & **8B**).

Determination of CABG patency is another indication for coronary artery MDCT. An accurate surgical history is vital as the course of bypass grafts whether from native arteries or venous can vary widely. The entire chest should be scanned in order to evaluate the complete course of an internal mammary artery (IMA). Coronary artery stent patency can also be assessed [23, 25]. 

Artifacts in CCTA, such as streaking and ‘blooming’ of calcified plaque, metallic implants such as stents and surgical clips, can result in overestimation of plaque burden, by partially obscuring the patent lumen of the artery. Beyond recognition of this phenomenon by an experienced interpreting physician, allowing one to correctly interpret the image data, post-processing tools, in various stages of current commercial release and development from all scanner manufacturers and many software vendors, can assist in reducing blooming artifacts caused by calcium and stent and bypass grafts by sharpening and edge enhancement algorithms.

### Cardiac Morphology & Function

High spatial resolution and low image noise achievable with MDCT makes this modality perfect for depicting normal and abnormal myocardium. The morphology of the heart, its chambers, arterial and venous connections, valves and the myocardium are clearly depicted. Functional analysis based on MDCT data correlates well with MRI functional analysis [26].

Software packages capable of cardiac functional analysis, i.e., estimation of ejection fraction, stroke volume and cardiac output are available from all scanner manufacturers and from several vendors that specialize only in post-processing software. Each uses a software program which is capable of calculating chamber volume approximation in the method based upon discs. Sources of error in left ventricle (LV) functional measurements can be remedied if the number of slices of the LV and the reconstruction temporal resolution is optimized [27].

The thickness and intermediate HU attenuation of the myocardium are normally uniform. Scarred myocardium, perhaps damaged by infarction, is seen as myocardial wall thinning and replacement of normal myocardium by fibrotic tissue which is lower attenuation compared to the surrounding myocardium [11, 23] Fig. (**9A** & **9B**).

Wall motion abnormalities can be detected when the heart has been scanned using retrospective ECG-gating because the entire cardiac cycle will have been imaged. Post-processing programs can then reconstruct numerous segments of the R-R interval which when put into cine or movie loop functions will simulate cardiac motion. When viewed in standard cardiac imaging planes, one can assess myocardial wall motion just as is done with echocardiography and MRI [23, 28].

The morphology of the heart can be further assessed with MDCT; the shape of cardiac chambers, defects in the wall or septae [patent foramen ovale (PFO), atrial septal defect (ASD) and ventricular septal defect (VSD)] and other wall abnormalities such as thickening or aneurysm are clearly depicted with MDCT [23] Fig. (**10A** & **10B**).

### Valve Function

The atrioventricular and the ventriculoarterial valves are exquisitely shown using MDCT. Since it is important to view the anatomy of the valve in systole and diastole as well as while the valve is opening and closing, retrospective ECG-gating is mandatory when examining a valve. Thorough examination of a valve includes reconstruction of the image data in the plane of each valve such that the leaflet motion and the excursion and apposition of leaflets may be assessed in planar and 3D cine and still frame format [23, 29]. Though spatial resolution is greater with CT, compared to MRI, the relatively low temporal resolution of 64 channel CCTA may fail to show the valve accurately in end systole and end diastole and flow data are not available.

Disease processes that are easily identified include atherosclerotic thickening and calcification of leaflets, vegetations, thrombus and tumor, as well as functional assessment of the valves, recognizing stenosis, incompetence and leaflet malformation such as bicuspid aortic valve [30] Fig. (**11A** & **11B**).

### Cardiomyopathy

Although not yet clinically proven, it may be possible to identify the presence of dilated, hypertrophic and infiltrative myocardial disease on the basis of HU attenuation, myocardial thickening and wall motion abnormalities. Functional assessment of wall motion abnormalities and enhancement of the myocardium may contribute to diagnosis of these disease processes [31-33].

### Myocardial Perfusion

Regional and focal areas of relatively low attenuation compared to the normal myocardium, which correspond to vascular territories, have been reported as presumed myocardial perfusion defects, raising interest in the possibility of designing cardiac imaging protocols to evaluate myocardial perfusion and enhancement [34, 35].

Faster scanning protocols and true volumetric data acquisition increase the likelihood that reliable perfusion data and perhaps myocardial viability imaging of the myocardium will be available to anyone who owns a modern, at least 64 MDCT and understands the physics of acquiring such images.

### Pulmonary Vein Imaging

Prior to pulmonary vein ablation procedures MDCT is fundamental in order to provide precise anatomic knowledge of the widely variant anatomy of the venous ostium, branching pattern and the shape and to exclude atrial or atrial appendage thrombi Fig. (**12**). The accurate anatomic information can be integrated with angiographic image data to increase confidence of catheter placement and can decrease the time required to perform the ablation procedure. Non-ECG-gated MDCT is almost always adequate when imaging the pulmonary vein anatomy with 64 channel or greater MDCT. This is especially true in patients with arrhythmia, a contraindication for ECG-gating. Although use of ECG-gating could add information regarding the coronary artery anatomy, coronary artery anatomy and superior spatial resolution of ECG-gated scans is not essential [11, 36]. By performing a non-ECG-gated exam, the patient is spared up to 80% of the radiation dose. Exposing your patient unnecessarily to the radiation dose of ECG gated cardiac CT, just because the images are ‘prettier’ is irresponsible if the spatial resolution is not absolutely required.

Following pulmonary vein catheter ablation procedures MDCT is essential for imaging of rare complications such as atrioesphageal fistula (1%) [37] and for those not so rare; pulmonary vein ostial stenosis. Up to 28% of patients have been found to have stenosis of at least one pulmonary vein after ablation [38]. Therefore, a reliable diagnostic imaging study is needed for long-term follow-up. MDCT fills that void, especially in patients with a poor acoustic window and those for whom trans-esophageal echocardiography is difficult, as it is non-invasive, imparts a low radiation dose if prospective or a non ECG-gated technique are employed and exquisitely reveals the pulmonary vein anatomy [36, 37, 39]. MDCT of pulmonary vein anatomy can be accurately depicted with non-gated MDCT even when using 16 detector channels [40].

### Thoracic Aorta & Pulmonary Arteries

Even in limited anatomical assessment of the heart, segments of the ascending and descending thoracic aorta are included in coronary artery CTA images. Secondary findings such as dilatation of the ascending aorta due to a bicuspid aortic valve, aneurysm or dissection due to atherosclerosis or other vascular disease, pulmonary hypertension or pulmonary embolus are potential diagnoses to recognize. When these secondary diagnoses are seen, a complete thoracic CT is warranted. In the case of aneurysm and dissection, the entire abdomen and pelvis may need to be included in follow-up studies [41] Fig. (**13A** & **13B**).

### The Mediastinum & Upper Abdomen

Beyond the cardiac structures, important findings will be included in the imaging field of view. At a minimum, image reconstruction to include the entire chest wall, within the z-axis of the scan should be performed. This said, currently there are limitations on the field of view (FOV) which limit the reconstruction of prospective ECG-gated sequences. Based upon scan type and manufacturer, the reconstruction FOV may be limited to the scanned FOV rather than including the anatomy outside that FOV Fig. (**14A** & **14B**). Mediastinal and axillary lymph nodes, the trachea, esophagus, pulmonary parenchyma and upper abdominal viscera are also included along with the images of the heart. Each structure can show additional diagnoses or clues to alternative diagnoses [41, 42].

## CONCLUSION

The rapid and continued development of MDCT technology brings an exciting twist to cardiac imaging. Enhancing patient care by improved visualization and understanding of coronary artery anatomy, valve performance and myocardial function and perfusion will continue most efficiently if the end user fully understands the imaging modality and how to get the most out of each manufacturer’s contribution to the field. 

The ultimate challenge is to use the image data to make accurate diagnoses of cardiac and non-cardiac findings to benefit the patient. A correct and complete diagnostic examination of the coronary arteries, cardiac chambers and valves depends upon consideration of all types of cardiac pathology. One can only see what one looks for: cardiac and noncardiac pathology that is not considered because of a pre-determined expectation of findings, either based upon clinical knowledge or incomplete familiarity with cardiac MDCT will result in a disservice to the patient and will limit future development of the imaging modality. The number of detector channels is not the operative key; it is the relationship of radiologists and cardiologists working together which ultimately will achieve the goal of evaluating the appropriateness of cardiac MDCT and reaching accurate and complete diagnostic interpretation. This collaboration will drive the direction of imaging technology.

## Figures and Tables

**Fig. (1):**
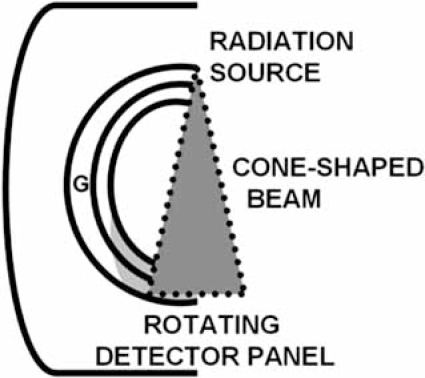
Anatomy of a multidetector CT scanner. A single radiation source and the multi channel detector are contained within the scanner gantry (G) 180 degrees from each other. A cone-shaped beam of radiation passes through the patient to the detector. The table on which the patient lies moves through the gantry during the scan.

**Fig. (2):**
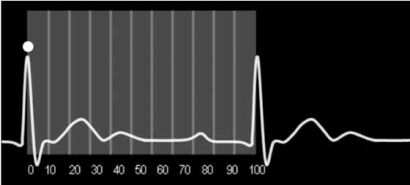
The R-R interval can theoretically be divided into 100 segments for the purposes of cardiac CT.  The first R wave (circle) is the 0% time point at end diastole; the next R wave is the 100% time point of the first cardiac cycle and the 0% time point of the next cardiac cycle.

**Fig. (3):**
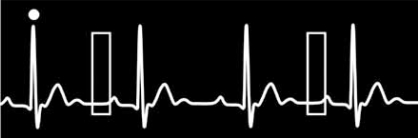
Prospective gating. Scan acquisition occurs at a user defined time point after the R wave (circle). The radiation source is active only during a short segment of the R-R interval (rectangle).

**Fig. (4):**
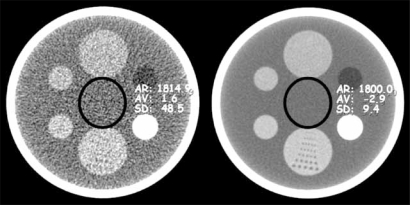
Image noise. The image on the left has a higher degree of image noise, measured as a standard deviation in the range of HU of 48 HU. On the right, the image has a more homogeneous appearance; less noise, which is measured as 9.4 HU. Notice also that noise affects spatial resolution; definition of the perforated plate at the 6 o'clock position of the phantom on the left with greater image noise are nearly imperceptible.

**Fig. (5):**
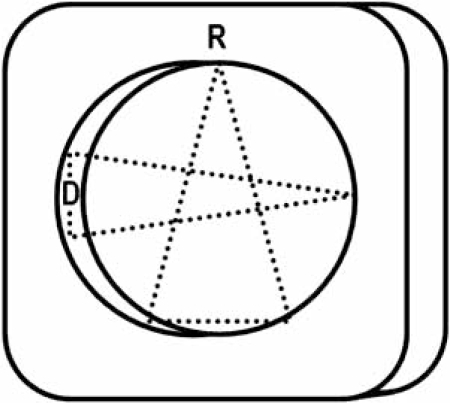
Dual source MDCT. Two radiation sources and two detector panels are oriented at 90 degrees from each other. A cone-shaped beam of radiation passes from each source, through the patient to each detector. The table on which the patient lies moves through the gantry during the scan.

**Fig. (6):**
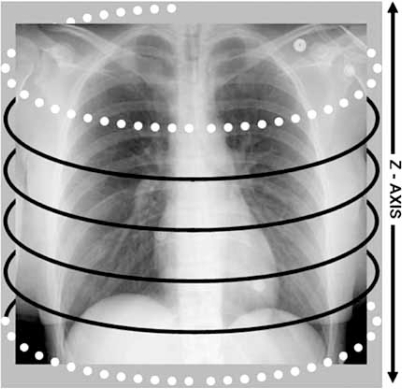
The portion of the scan referred to as over-scanning occurs at the beginning and end or at the edges of the scan (dotted white lines). Black lines represent the extent of radiation that is imparted in the scan to create the image.

**Fig. (7A):**
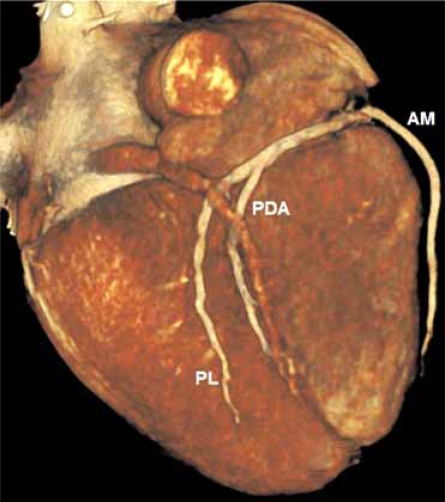
Looking at the inferior surface of the heart, this 3D surface rendered image show the distal branches of the dominant right coronary artery; acute marginal (AM), posterior descending (PDA) and a posterolateral branch (PL).

**Fig. (7B):**
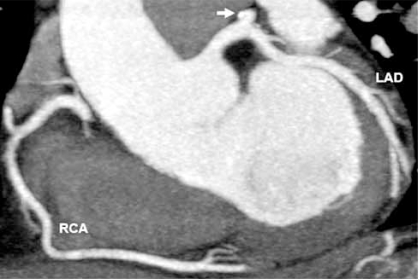
Normal left anterior descending (LAD) and right coronary (RCA) arteries are shown in a 2D curved planar reformatted image from a retrospectively ECG-gated MDCT data set.  A punctuate focus of calcification is in the proximal left circumflex artery (arrow).

**Fig. (7C):**
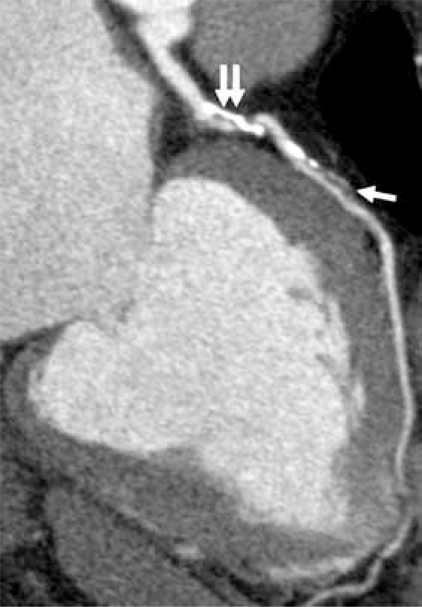
Calcified (double arrow) and non-calcified (single arrow) atherosclerotic plaque causes narrowing of the proximal LAD shown in this 2D curved planar reformatted image from a retrospectively ECG-gated MDCT data set.

**Fig. (8A):**
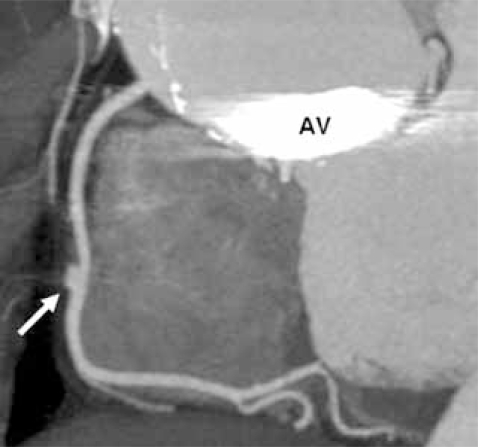
A coronal oblique 2D reformatted image shows an aneurysm of the mid RCA in this patient with Marfan syndrome.  Note the prosthetic aortic valve (AV) and dilated ascending aorta.

**Fig. (8B):**
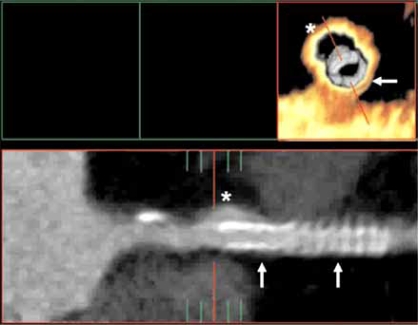
Although motion artifact is seen, an endovascular and layout 2D curved reformat view of the proximal LAD show an aneurysm of the LAD (asterisk) which formed during placement of two coronary artery stents (arrows).

**Fig. (9A):**
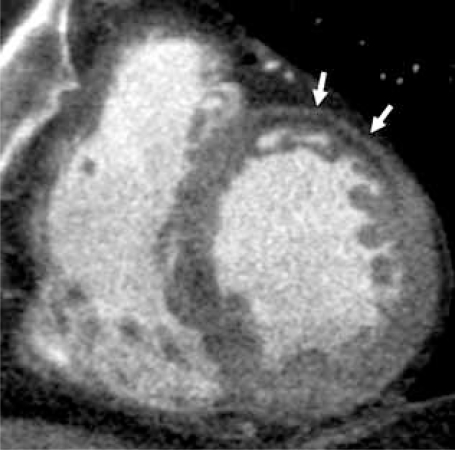
Shown in a short axis view, linear low attenuation fibrotic tissue (white arrows) has replaced myocardium of the anterior wall of the left ventricle in this patient with known history of thrombois of a LIMA CABG.

**Fig. (9B):**
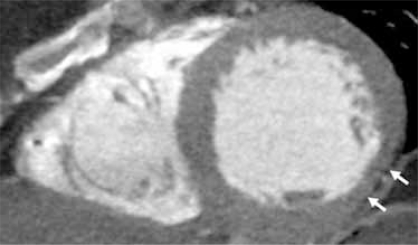
Shown in a short axis view, thinning of the inferolateral LV wall is seen (white arrows).  The myocardium is visually normal attenuation, but measured 8-15 HU less than the HU attenuation of the septal, anterior and inferior walls.

**Fig. (10A):**
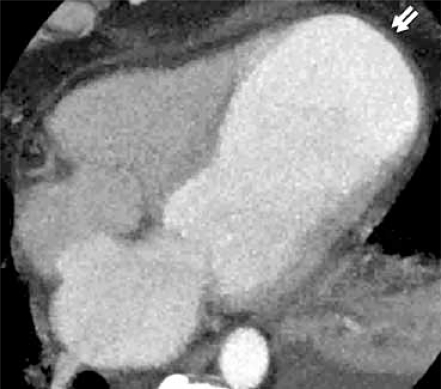
In a 4 chamber (4CH) view the LV apex is aneurismal in this patient who experienced MI in the LAD vascular distribution 30 days prior to this study.

**Fig. (10B):**
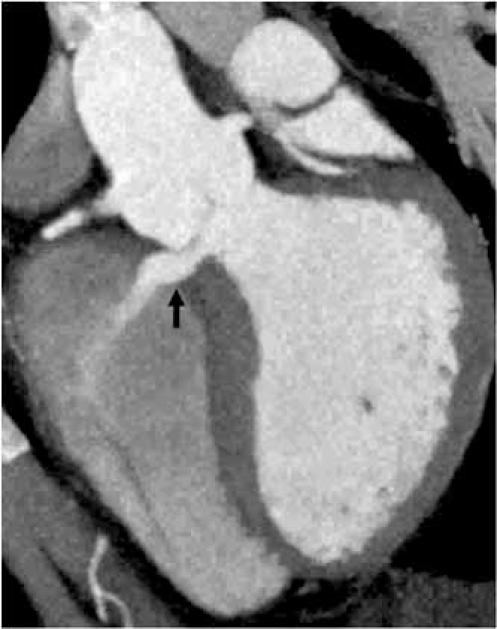
A curved 2D reformatted image of the interventricular septum shows a tiny membraneous ventricular septal defect (VSD) (black arrow).

**Fig. (11A):**
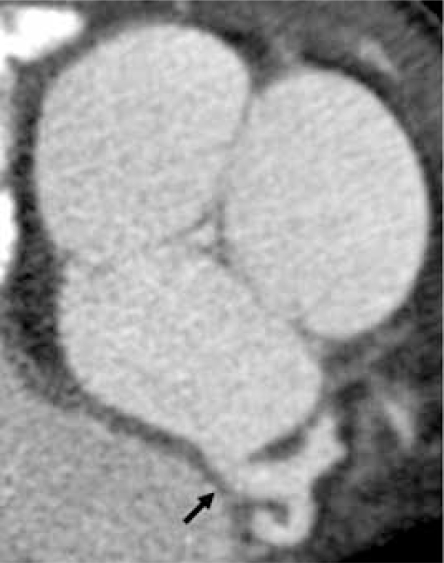
The closed aortic valve is show in an axial oblique 2D maximum intensity projection (MIP) image.  The left main coronary artery origin is seen arising from the left coronary cusp (black arrow).

**Fig. (11B):**
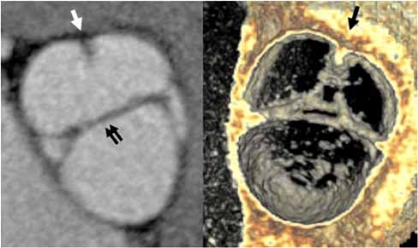
A bicuspid aortic valve is shown in closed position (double arows) in an axial oblique 2D MIP and an endovascular 3D view, looking into the sinuses of valsalva.  The right and left cusps are partially fused (single arrows).

**Fig. (12):**
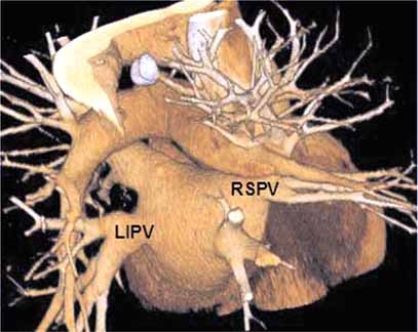
The pulmonary veins are exquisitely shown in this 3D surface rendered image of the heart.  Viewed from a posterior, the left inferior pulmonary vein (LIPV) and the right superior pulmonary vein (RSPV) are labeled.

**Fig. (13A):**
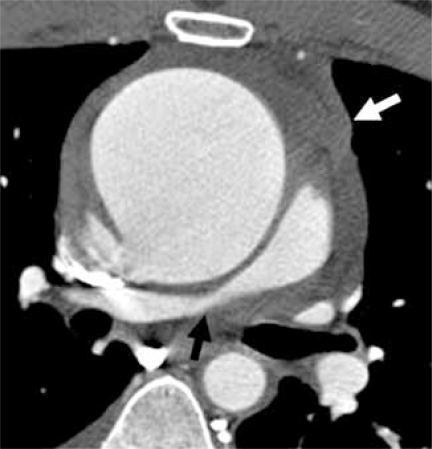
Axial view of the superior mediastinum shows marked dilation of the ascending aorta resulting in extrinsic compression of the right pulmonary artery (black arrow) and intermediate attenuation hemorrhage (white arrow) indicating leakage from the ruptured ascending aorta in this emergency room patient with chest pain.

**Fig. (13B):**
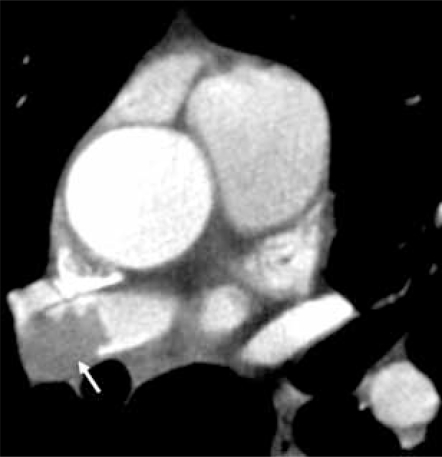
Axial view of the superior mediastinum shows a filling defect in the right pulmonary artery (white arrow) representing pulmonary embolus in this emergency room patient with chest pain.

**Fig. (14A):**
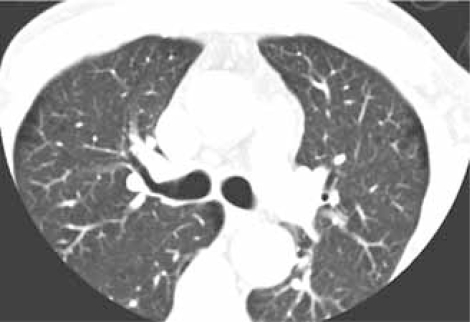
A limited field of view (FOV) is utilized to improve spatial resolution during scanning of the coronary arteries, resulting in eliminating pulmonary parenchyma and parts of the chest wall from the image.

**Fig. (14B):**
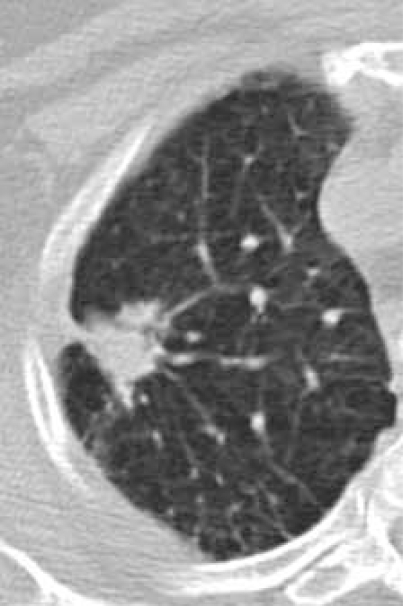
Depending on the scan protocol, image data may be reconstructed to include the tissues outside of the scan FOV. In this instance, a spiclutaed, 1 cm lung cancer is discovered.

**Table 1.** Comparison of 4 Major Manufacturer Scanner Options for Cardiac MDCT

	GENERAL ELECTRIC^*^		PHILIPS^*^		SIEMENS^*^			TOSHIBA^*^		
NUMBER OF DETECTOR CHANNELS	64	128	64	256	64	64	128	64	256	320
MODEL NAME	Light speed VCT XT	LightSpeed CT750 HD	Brilliance 64^*^	Brilliance iCT^*^	SOMATOM Definiton Dual Source CT^*^	SOMATOM Definition^*^	SOMATOM Definition AS^*^	Aquilion 64	Aquilon 256	AquilionONE
SPATIAL RESOLUTION	.35 mm	in development	0.34 mm - up to 24lp resolution	24 lp/cm - 13 lp/cm resolution	0.33 mm	0.33 mm	0.33 mm	0.35 mm	0.35 mm	0.35 mm
TEMPORAL RESOLUTION	175 msec	in development	165 msec	100 msec	83 ms independent of the heart rate down to 42 ms using 2 segment reconstruction	down to 83 ms	down to 75 ms	175 ms	200 ms	350 ms
GANTRY ROTATION SPEED	0.35 sec	0.33 sec	0.33 sec	0.27 sec	0.33, 0.5 & 1.0 sec	0.33 sec	0.3 sec	0.35 sec	0.4 sec	0.35 sec
COLLIMATION	64 x 0.625 mm	64 x 0.625	64 x 0.625 mm	256 x 0.625 mm; 2 - 128 x 0.625 - 1.25 mm fused	Dual Source, dual power modes: 2 x 64 x 0.6 mm Single source spiral modes: 64 x 0.6 mm	64 x 0.6 mm	128 x 0.6 mm, 64 x 0.6 mm	64 x 0.5 mm	256 x 0.5 mm	320 x 0.5 mm
DETECTOR COVERAGE	40 mm	80 mm	40 mm	80 mm	28.8 mm	28.8 mm	75 mm	32 mm	128 mm	160 mm
DUAL SOURCE	NO	NO	NO	NO	YES	NO	NO	NO	NO	NO
DUAL ENERGY	NO	NO	NO	NO	YES	NO	NO	NO	NO	NO
DOSE SAVING SOFTWARE	SnapShot pulse^*^	in development	DoseWise^*^; Step & Shoot Cardiac^*^	Step & Shoot Cardiac^*^, DoseWise^*^ philosophy; DoseRight ACS^*^; DoseRight D-DOM^*^; DoseRight Z-DOM^*^; IntelliBeam^*^ filters;Wedges; Eclipse DoseRight^*^ overcomes overscan	CARE applications - CARE Dose4D^*^	CARE applications - CARE Dose4D^*^	CARE applications - CARE Dose4D^*^	^SURE^Cardio Prospective^*^	^SURE^Cardio Prospective^*^	^SURE^Cardio Prospective^*^
% DOSE REDUCTION	up to 83%	up to 83%	DoseRight^*^ - up to 80%; Step & Shoot Cardiac^*^ - up to 45%	Beta production - estimates no decrease in dose compared to prospectively gated 64 MDCT cardiac CTA. Bow tie filters are investigational; may allow 10% - 20% dose reduction	UFC Detector^*^ - up to 30% SureView^*^ - up to 20% CARE Filter^*^ - up to 25% CARE Dose4D^*^ - up to 68%	UFC Detector^*^ - up to 30% SureView^*^ - up to 20% CARE Filter^*^ - up to 25% CARE Dose4D^*^ - up to 68% Adaptive ECG-Pulsing^*^ - up to 50 %	UFC Detector^*^ - up to 30% SureView^*^ - up to 20% CARE Filter^*^ - up to 25% CARE Dose4D^*^ - up to 68% Adaptive Dose Shield^*^ - up to 20% Adaptive ECG-Pulsing^*^ - up to 50%			Beta production - estimates no decrease in dose compared to prospectively gated 64 MDCT cardiac CTA. Potential dose reduction of up to 40% with investigational filtration techniques.

Sources: accessed May 25, 2008

http://www.gehealthcare.com/usen/ct/index.html

http://www.medical.philips.com/main/products/ct/

http://www.medical.siemens.com

http://www.medical.toshiba.com/Products/CT/

**Table 2.** Effective Radiation Dose Values for Common Diagnostic Imaging Procedures and Background Radiation (mSV)

Chest radiograph	0.02
Roundtrip airplane NY to LA	0.03
Mammogram	0.13
Brain CT	1-2
Chest CT	5-7
Coronary artery calcium EBCT	0.6
Coronary artery calcium CT – MDCT prospective ECG-gating	1-3
Coronary CTA – prospective ECG-gating dose modulation (80 kV)	2-4
Background radiation in the US	3.6 – (range 1-10)
Conventional coronary artery angiography	2-10
Rubidium PET stress test	4.9
Coronary CTA – retrospective ECG-gating	10-15 mSv
Sestamibi SPECT nuclear stress test	12-23
	
Cardiologist dose – conventional coronary artery angiography	0.1-5*
Nurse dose – conventional coronary artery angiography	0.01-0.5*

* estimated annual dose

mSv milli Sieverts – effective radiation dose

References

[11] Fishman E. Cardiac CT: Where are we today and where are we going? Applied Radiol Supp. December 2006; 5-8.

[13] McCollough CH et. al. Dose performance of a 64-channel dual-source CT scanner. Radiol (2007); 243(3): 775-784.
